# Supplementary material for: Pyruvate Dehydrogenase Kinase 1 inhibition mediated oxidative phosphorylation enhancement in cartilage promotes osteoarthritis progression
Source: BMC Musculoskelet Disord. 2023 Jul 20;24:597. doi: 10.1186/s12891-023-06585-6 (PMC10357736; doi:10.1186/s12891-023-06585-6)
Supplement: Supplementary file 1 — Additional file 1. [file 12891_2023_6585_MOESM1_ESM.zip › MF.Gsea.1653627316242/GOMF_OLFACTORY_RECEPTOR_ACTIVITY.html]

Details for gene set GOMF\_OLFACTORY\_RECEPTOR\_ACTIVITY[GSEA]

|  || Dataset | 55457\_collapsed\_to\_symbols.cls.cls #OA\_versus\_normal.cls.cls #OA\_versus\_normal\_repos |
| Phenotype | cls.cls#OA\_versus\_normal\_repos |
| Upregulated in class | normal |
| GeneSet | GOMF\_OLFACTORY\_RECEPTOR\_ACTIVITY |
| Enrichment Score (ES) | -0.38033193 |
| Normalized Enrichment Score (NES) | -1.3849413 |
| Nominal p-value | 0.06678082 |
| FDR q-value | 0.28586698 |
| FWER p-Value | 1.0 |
Table: GSEA Results Summary

  

Fig 1: Enrichment plot: GOMF\_OLFACTORY\_RECEPTOR\_ACTIVITY      
 Profile of the Running ES Score & Positions of GeneSet Members on the Rank Ordered List

  

| SYMBOL | TITLE | RANK IN GENE LIST | RANK METRIC SCORE | RUNNING ES | CORE ENRICHMENT || 1 | OR2J2 | olfactory receptor family 2 subfamily J member 2 [Source:HGNC Symbol;Acc:HGNC:8260] | 1367 | 0.448 | -0.0637 | No |
| 2 | OR7A5 | olfactory receptor family 7 subfamily A member 5 [Source:HGNC Symbol;Acc:HGNC:8368] | 1943 | 0.368 | -0.0735 | No |
| 3 | OR1E3 | olfactory receptor family 1 subfamily E member 3 [Source:HGNC Symbol;Acc:HGNC:8191] | 2120 | 0.347 | -0.0542 | No |
| 4 | OR11A1 | olfactory receptor family 11 subfamily A member 1 [Source:HGNC Symbol;Acc:HGNC:8176] | 3121 | 0.238 | -0.1094 | No |
| 5 | OR12D2 | olfactory receptor family 12 subfamily D member 2 [Source:HGNC Symbol;Acc:HGNC:8178] | 3191 | 0.232 | -0.0927 | No |
| 6 | OR2B2 | olfactory receptor family 2 subfamily B member 2 [Source:HGNC Symbol;Acc:HGNC:13966] | 3815 | 0.175 | -0.1246 | No |
| 7 | OR1D5 | olfactory receptor family 1 subfamily D member 5 [Source:HGNC Symbol;Acc:HGNC:8186] | 4113 | 0.145 | -0.1339 | No |
| 8 | OR10H1 | olfactory receptor family 10 subfamily H member 1 [Source:HGNC Symbol;Acc:HGNC:8172] | 4167 | 0.141 | -0.1246 | No |
| 9 | OR1A2 | olfactory receptor family 1 subfamily A member 2 [Source:HGNC Symbol;Acc:HGNC:8180] | 4314 | 0.127 | -0.1239 | No |
| 10 | OR52A1 | olfactory receptor family 52 subfamily A member 1 [Source:HGNC Symbol;Acc:HGNC:8318] | 4655 | 0.100 | -0.1409 | No |
| 11 | OR2S2 | olfactory receptor family 2 subfamily S member 2 [Source:HGNC Symbol;Acc:HGNC:8276] | 5152 | 0.059 | -0.1738 | No |
| 12 | OR12D3 | olfactory receptor family 12 subfamily D member 3 [Source:HGNC Symbol;Acc:HGNC:13963] | 5159 | 0.059 | -0.1687 | No |
| 13 | OR10J1 | olfactory receptor family 10 subfamily J member 1 [Source:HGNC Symbol;Acc:HGNC:8175] | 5255 | 0.052 | -0.1712 | No |
| 14 | OR3A2 | olfactory receptor family 3 subfamily A member 2 [Source:HGNC Symbol;Acc:HGNC:8283] | 6097 | -0.012 | -0.2354 | No |
| 15 | OR10H3 | olfactory receptor family 10 subfamily H member 3 [Source:HGNC Symbol;Acc:HGNC:8174] | 6287 | -0.026 | -0.2476 | No |
| 16 | OR1A1 | olfactory receptor family 1 subfamily A member 1 [Source:HGNC Symbol;Acc:HGNC:8179] | 6444 | -0.038 | -0.2561 | No |
| 17 | OR2B6 | olfactory receptor family 2 subfamily B member 6 [Source:HGNC Symbol;Acc:HGNC:8241] | 6524 | -0.044 | -0.2580 | No |
| 18 | OR1D2 | olfactory receptor family 1 subfamily D member 2 [Source:HGNC Symbol;Acc:HGNC:8183] | 6577 | -0.048 | -0.2575 | No |
| 19 | OR51E2 | olfactory receptor family 51 subfamily E member 2 [Source:HGNC Symbol;Acc:HGNC:15195] | 6898 | -0.072 | -0.2756 | No |
| 20 | OR1F2P | olfactory receptor family 1 subfamily F member 2 pseudogene [Source:HGNC Symbol;Acc:HGNC:8196] | 7244 | -0.099 | -0.2930 | No |
| 21 | OR1G1 | olfactory receptor family 1 subfamily G member 1 [Source:HGNC Symbol;Acc:HGNC:8204] | 7387 | -0.109 | -0.2937 | No |
| 22 | OR1F1 | olfactory receptor family 1 subfamily F member 1 [Source:HGNC Symbol;Acc:HGNC:8194] | 7409 | -0.111 | -0.2847 | No |
| 23 | OR7C2 | olfactory receptor family 7 subfamily C member 2 [Source:HGNC Symbol;Acc:HGNC:8374] | 7443 | -0.114 | -0.2765 | No |
| 24 | OR2W1 | olfactory receptor family 2 subfamily W member 1 [Source:HGNC Symbol;Acc:HGNC:8281] | 8780 | -0.215 | -0.3599 | Yes |
| 25 | OR2H1 | olfactory receptor family 2 subfamily H member 1 [Source:HGNC Symbol;Acc:HGNC:8252] | 8851 | -0.221 | -0.3444 | Yes |
| 26 | OR2H2 | olfactory receptor family 2 subfamily H member 2 [Source:HGNC Symbol;Acc:HGNC:8253] | 9097 | -0.239 | -0.3407 | Yes |
| 27 | OR2F2 | olfactory receptor family 2 subfamily F member 2 [Source:HGNC Symbol;Acc:HGNC:8247] | 9316 | -0.257 | -0.3333 | Yes |
| 28 | OR1E1 | olfactory receptor family 1 subfamily E member 1 [Source:HGNC Symbol;Acc:HGNC:8189] | 9744 | -0.296 | -0.3383 | Yes |
| 29 | OR10H2 | olfactory receptor family 10 subfamily H member 2 [Source:HGNC Symbol;Acc:HGNC:8173] | 10006 | -0.322 | -0.3281 | Yes |
| 30 | OR2F1 | olfactory receptor family 2 subfamily F member 1 [Source:HGNC Symbol;Acc:HGNC:8246] | 10148 | -0.336 | -0.3071 | Yes |
| 31 | OR5I1 | olfactory receptor family 5 subfamily I member 1 [Source:HGNC Symbol;Acc:HGNC:8347] | 10616 | -0.385 | -0.3069 | Yes |
| 32 | OR7A17 | olfactory receptor family 7 subfamily A member 17 [Source:HGNC Symbol;Acc:HGNC:8363] | 10697 | -0.392 | -0.2758 | Yes |
| 33 | OR2J3 | olfactory receptor family 2 subfamily J member 3 [Source:HGNC Symbol;Acc:HGNC:8261] | 10789 | -0.404 | -0.2445 | Yes |
| 34 | OR7C1 | olfactory receptor family 7 subfamily C member 1 [Source:HGNC Symbol;Acc:HGNC:8373] | 10945 | -0.424 | -0.2163 | Yes |
| 35 | OR7A10 | olfactory receptor family 7 subfamily A member 10 [Source:HGNC Symbol;Acc:HGNC:8356] | 11448 | -0.496 | -0.2083 | Yes |
| 36 | OR3A1 | olfactory receptor family 3 subfamily A member 1 [Source:HGNC Symbol;Acc:HGNC:8282] | 11554 | -0.508 | -0.1682 | Yes |
| 37 | OR3A3 | olfactory receptor family 3 subfamily A member 3 [Source:HGNC Symbol;Acc:HGNC:8284] | 11603 | -0.516 | -0.1229 | Yes |
| 38 | OR2C1 | olfactory receptor family 2 subfamily C member 1 [Source:HGNC Symbol;Acc:HGNC:8242] | 12366 | -0.696 | -0.1160 | Yes |
| 39 | OR7E24 | olfactory receptor family 7 subfamily E member 24 [Source:HGNC Symbol;Acc:HGNC:8396] | 12451 | -0.725 | -0.0537 | Yes |
| 40 | OR10C1 | olfactory receptor family 10 subfamily C member 1 [Source:HGNC Symbol;Acc:HGNC:8165] | 12776 | -0.932 | 0.0096 | Yes |
Table: GSEA details [plain text format]

  

Fig 2: GOMF\_OLFACTORY\_RECEPTOR\_ACTIVITY      
 Blue-Pink O' Gram in the Space of the Analyzed GeneSet

  

Fig 3: GOMF\_OLFACTORY\_RECEPTOR\_ACTIVITY: Random ES distribution      
 Gene set null distribution of ES for **GOMF\_OLFACTORY\_RECEPTOR\_ACTIVITY**
